# Filtration performance, fit test and side effects of respiratory personal protective equipment following decontamination: Observations for user safety and comfort

**DOI:** 10.1371/journal.pone.0280426

**Published:** 2023-01-23

**Authors:** Nathalie Turgeon, Mélissa Pagé, Justin Robillard, Véronique Goulet, Ali Bahloul, Clothilde Brochot, Mohamed Nejib Saidi, Nathan Dumont-Leblond, Caroline Duchaine

**Affiliations:** 1 Centre de Recherche de l’Institut Universitaire de Cardiologie et de Pneumologie de Québec- Université Laval, Quebec City, Quebec, Canada; 2 Institut de Recherche Robert-Sauvé en Santé et en Sécurité du Travail, Montreal, Quebec, Canada; 3 Department of Building, Civil and Environmental Engineering, Concordia University, Montreal, Quebec, Canada; 4 Département de Biochimie, de Microbiologie et de Bio-informatique, Faculté des Sciences et de Génie, Université Laval, Quebec City, Quebec, Canada; 5 Canada Research Chair on Bioaerosols, Quebec City, QC, Canada; University of Patras, GREECE

## Abstract

**Objective:**

While facing personal protective equipment (PPE) shortages during the COVID-19 pandemic, several institutions looked to PPE decontamination and reuse options. This study documents the effect of two hydrogen peroxide treatments on filtration efficiency and fit tests as well as the side effects for volunteers after the decontamination of N95 filtering facepiece respirators (FFRs). We also propose an efficient and large-scale treatment protocol that allows for the traceability of this protective equipment in hospitals during PPE shortages.

**Methods:**

The effects of low-temperature hydrogen peroxide sterilization and hydrogen peroxide vapor (HPV) on two FFR models (filtration, decontamination level, residual emanation) were evaluated. Ten volunteers reported comfort issues and side effects after wearing 1h FFRs worn and decontaminated up to five times.

**Results:**

The decontamination process does not negatively affect FFR efficiency, but repeated use and handling tend to lead to damage, limiting the number of times FFRs can be reused. Moreover, the recommended 24-h post-treatment aeration does not sufficiently eliminate residual hydrogen peroxide. Prolonged aeration time increased user comfort when using decontaminated FFRs.

**Conclusions:**

HPV and low-temperature hydrogen peroxide sterilization seem to be appropriate treatments for FFR decontamination when the PPE is reused by the same user. PPE decontamination and reuse methods should be carefully considered as they are critical for the comfort and safety of healthcare workers.

## Introduction

In early 2020, the increased demand for personal protective equipment (PPE) spurred by the COVID-19 pandemic led to the risk of PPE shortages, including respiratory protection [1]. Potential shortages affected many countries including Canada, and more specifically, Quebec province. To reduce the demand for single-use equipment, several health services institutions considered the reuse of PPE by sterilizing between uses. Since reutilization is not recommended [2] and little was known about the effects of such practices, many safety concerns were raised. Therefore, the development of standardized PPE reuse methods became a necessity. Here, we studied N95 filtering facepiece respirator (FFR) decontamination and reuse.

Several factors must be considered in the decontamination and reuse of N95 FFRs. The filtration efficiency and fit of the equipment must be preserved to maintain N95 standard. N95 FFR decontamination should avoid leaving harmful residues while ensuring sufficient decontamination level [3]. The logistics of large-scale implementation of decontamination and reuse protocols, as well as the receptiveness of health care workers and strict compliance with the treatment process, are also important factors to consider.

Several approaches have been proposed for the decontamination and reuse of N95 FFRs: microwave oven [4], ethylene oxide [4], light or UV light [4–11], methylene blue [11], dry heat [7, 10, 12], autoclave [13–15], bleach [4, 10], and hydrogen peroxide [4, 7, 11, 16–21]. Bleach, ethylene oxide, and microwave oven has been shown to leave harmful residues or damage N95 FFRs [13, 22], while autoclaving and dry heat has had contradictory results [7, 10, 12–15]. This suggests that UV light, methylene blue and hydrogen peroxide are the most promising techniques [3, 23]. However, while a few studies have performed fit tests [17–19, 24–26], none have examined user comfort. Research institutes and agencies have published in-house protocols and validation results [27–30] which indicate effective filtration performance (filtration efficiency and pressure drop) after repetitive treatments and no detection of harmful residues. However, these protocols have been tested on a limited number of N95 FFR models [23]. Hydrogen peroxide treatments received Emergency Use Authorization from the FDA [31] and an interim order from Health Canada [2] since they were not previously intended for single-use PPE decontamination and reuse.

We conducted a small-scale study on the N95 FFR models that are used in our institute (Institut de Cardiologie et de Pneumologie de Québec-Université Laval), namely Moldex series1500 and 3M model 1870+. The treatments that are readily available at the institute are low-temperature hydrogen peroxide sterilization (Sterizone^®^ VP4 Sterilizer) and sterilization using a hydrogen peroxide vapor (HPV) generator (Bioquell L-4). Our goal was to test decontamination methods according to Health Canada guidelines [2] and to assess the feasibility of FFR decontamination in this setting. First, we used brand new FFRs to evaluate the decontamination efficiency of these treatments and the effect on filtration performance. Other masks were worn by volunteers and repeatedly treated to assess structural integrity via fit tests. Their level of comfort while using the N95 FFRs was also recorded. Hydrogen peroxide emanation was measured when volunteers first reported adverse effects when wearing the treated FFRs and modifications were made to the original protocol. Our study proposes a novel approach by including feedback from human participants to guide implementation practices.

## Materials and methods

### FFR decontamination treatments

New Moldex series 1500 N95 FFRs (molded, available in 5 sizes, nonadjustable nose bridge) and 3M model 1870+ FFR (folded in 3 parts, one size, adjustable nose bridge) were examined in this study. Low-temperature hydrogen peroxide sterilization treatment was performed in a Sterizone VP4 Sterilizer (TSO_3_, Québec, Canada) using the single preset cycle, identified as the “N95 Respirator Decontamination Cycle” in the Food and Drug Administration (FDA) Emergency Use Authorization. This sterilizer uses a combination of vaporized hydrogen peroxide and ozone and is intended for use in the terminal sterilization of cleaned, rinsed, and dried metal and nonmetal reusable medical devices in health care facilities. Sterizone^®^ BI+ Self-contained Biological Indicators (TSO_3_, Québec, Canada) (10^6^ spores of *Geobacillus stearothermophilus*) in process challenge devices were used to monitor the sterilizer cycles.

HPV treatment was performed using a Bioquell L-4 (Bioquell, Ecolab solution, PA, USA) in a 40 m^3^ room. The Bioquell L-4 is intended for the disinfection of rooms, materials, and enclosures using vaporized hydrogen peroxide. The HPV treatment cycle was as follows: 600 s of conditioning, gassing at an injection rate of 5.0 g/min for 3200 s, dwell phase injection at a rate of 2.0 g/min for 1200 s, and aeration for 1800 s. Chemical and biological disinfection indicators (HPV-Bi, Bioquell, PA, USA) were added to the chamber to monitor the disinfection process. Self-contained biological indicators (10^6^ spores of *Geobacillus stearothermophilus*) were also placed between 2 FFRs sealed with tape (6 per cycle).

The effects of Sterizone VP4 and Bioquell L-4 on FFRs were evaluated using: filtration efficiency testing, hydrogen peroxide emission measurement, and fit testing with volunteers. With Bioquell L4 decontamination control was performed using six self-contained biological indicators (10^6^ spores of *Geobacillus stearothermophilus*) placed between 2 FFRs sealed with tape. With Sterizone VP4 the sterilizer was physically monitored and controlled by software that triggered pre-set alarms if critical parameters were not met. A process challenge device (Test Pack) containing a self-contained biological indicator (10^6^ spores of *Geobacillus stearothermophilus*) and a Type 1 Chemical Indicator was used in the sterilization pouches.

### FFR filtration performance

**Fig 1:**
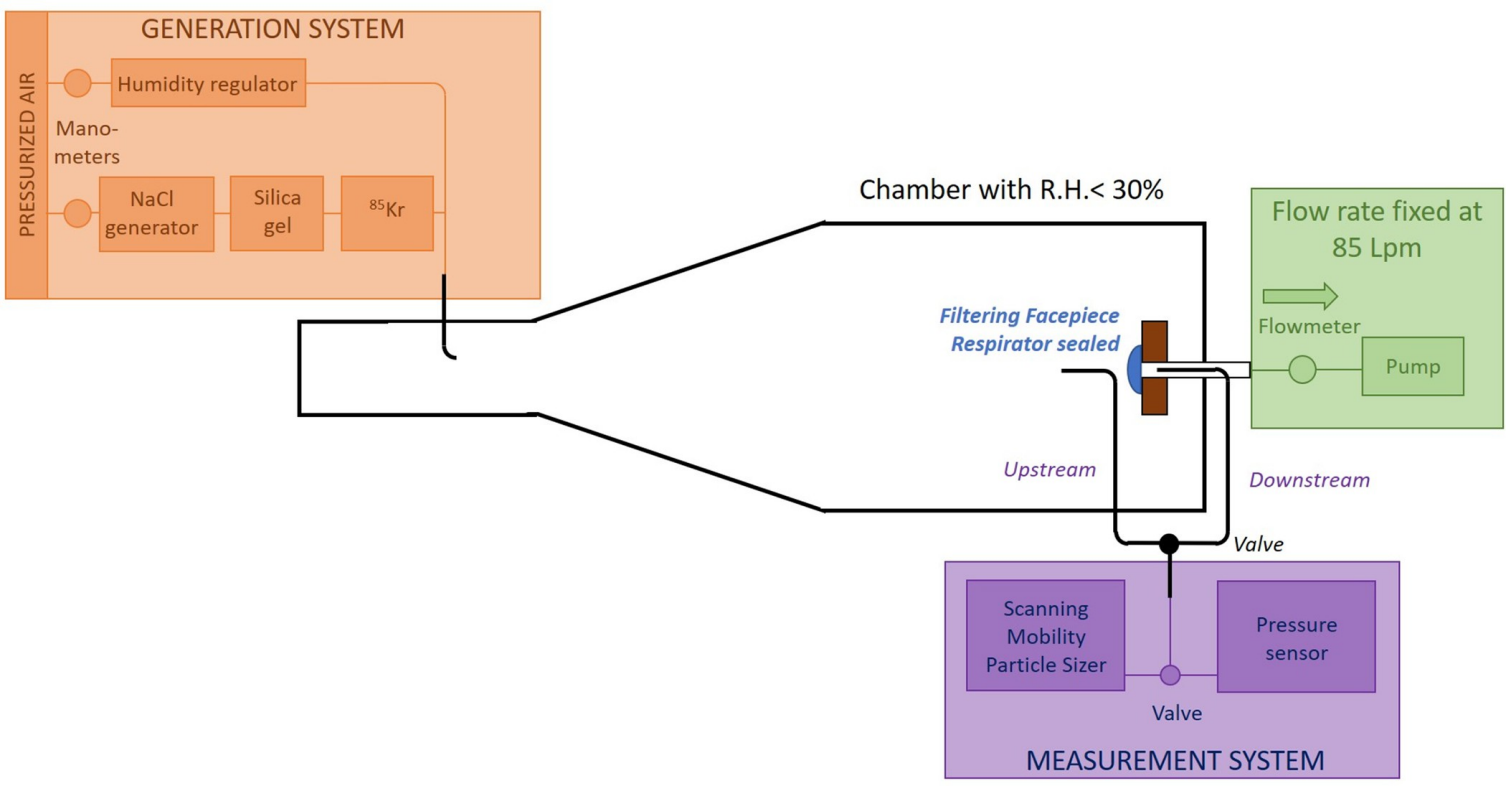
Schematic of the experimental setup used to measure the performance of the FFR. The elements labeled in orange were used for the generation of NaCl submicron particles. These particles were then released into the test chamber, and filtration performance was calculated using the measuring devices labeled in purple.

The filtration performance of FFRs was tested following treatment using the experimental setup presented in Fig 1 [32, 33]. Untreated FFRs were used as controls. The test chamber was designed to provide a controlled environment, where the flow rate through of the FFR was constant. Aerosol generation remained homogeneous and constant throughout the entire experiment. The tested FFRs were installed on a support plate and sealed with adhesive tape. To ensure that there are no leaks, the pressure drop is measured during the adjustment of the FFR on the plate. The greatest pressure drop is then measured during the best position and should remain stable.

The aerosol used consisted of NaCl particles that ranged from 20 nm to 600 nm in diameter (median around 70 nm). The aerosol was generated using a 6-jet Collison Nebulizer (CN2425 BGI Inc., Waltham, MA, USA) filled with a NaCl 0.1% (v/v) solution. The charges of aerosols were neutralized (Boltzmann equilibrium) using a Kr-85 Aerosol Neutralizer (3054A, TSI Inc., Shoreview, MN, USA). The particles were dried, diluted with pressurized air, and injected into the test chamber. Relative humidity measured in the chamber was below 30%.

A constant flow rate of 85 L/min was maintained in the chamber according to NIOSH procedures [34]. Two sample probes (of the same length) were used to collect the aerosol samples upstream and downstream of the FFRs. The same two probes were also used to measure the pressure drop across the FFRs using a FLUKE 922 pressure sensor (Fluke corp., Everett, WA, USA), which measures pressure at a range of ± 40 mbar, with a reading accuracy of ± 1%. The pressure drop is calculated according to Eq (1).


$$\Delta p=p_{upstream}-p_{downstream}$$ (1)

The FFR’s filtration efficiency E is given as a function of particle concentrations both downstream and upstream of the FFR, as expressed by Eq (2).


$$E=1-P=1-\frac{C_{dowstream}}{C_{upstream}}$$ (2)

The aerosol concentration by size was measured upstream and downstream of the FFR using a Scanning Mobility Particle Sizer (SMPS) (TSI 3080, TSI 3081, TSI 3087, TSI 3775, TSI Inc., Shoreview, MN, USA).

Particle size distributions were measured according to the following sequence: upstream (three scans), downstream (three scans), and finally upstream (two scans) again. The stability of the particle size distributions during the experiment was verified by comparing the two sets of upstream measurements. Only the minimum filtration efficiency was reported, according to the particle sizes that were least captured. The pressure drop and flow rate were measured before and after particle size distribution readings. The FFR was then removed and another FFR was tested following the same protocol. This paper presents the mean and the standard deviation of the samples (N = 3 to 8) for each N95 FFR.

### FFR treatment and reuse by volunteers

This project was conducted at IUCPQ-UL and approved by the IUCPQ-UL ethics committee (2021–3453 21917). Ten volunteers from the research center of IUCPQ-UL took part in this study (5 males, 5 females). Inclusion criteria were: working in the research center of IUCPQ-UL, no COVID-19 related symptoms, no contact with SARS-CoV-2 positive person, no contact with patients hospitalized at the IUCPQ-UL. Participants signed an Ethics Committee-approved consent form. No participant was minor. Volunteers were permitted to test more than 1 type of FFR. Volunteers that were not familiar with N95 passed a fit test before the study to identify the appropriate FFR. In the meantime they were instructed how to adjust, wear and remove FFRs. One volunteer left midway through the study.

**Fig 2:**
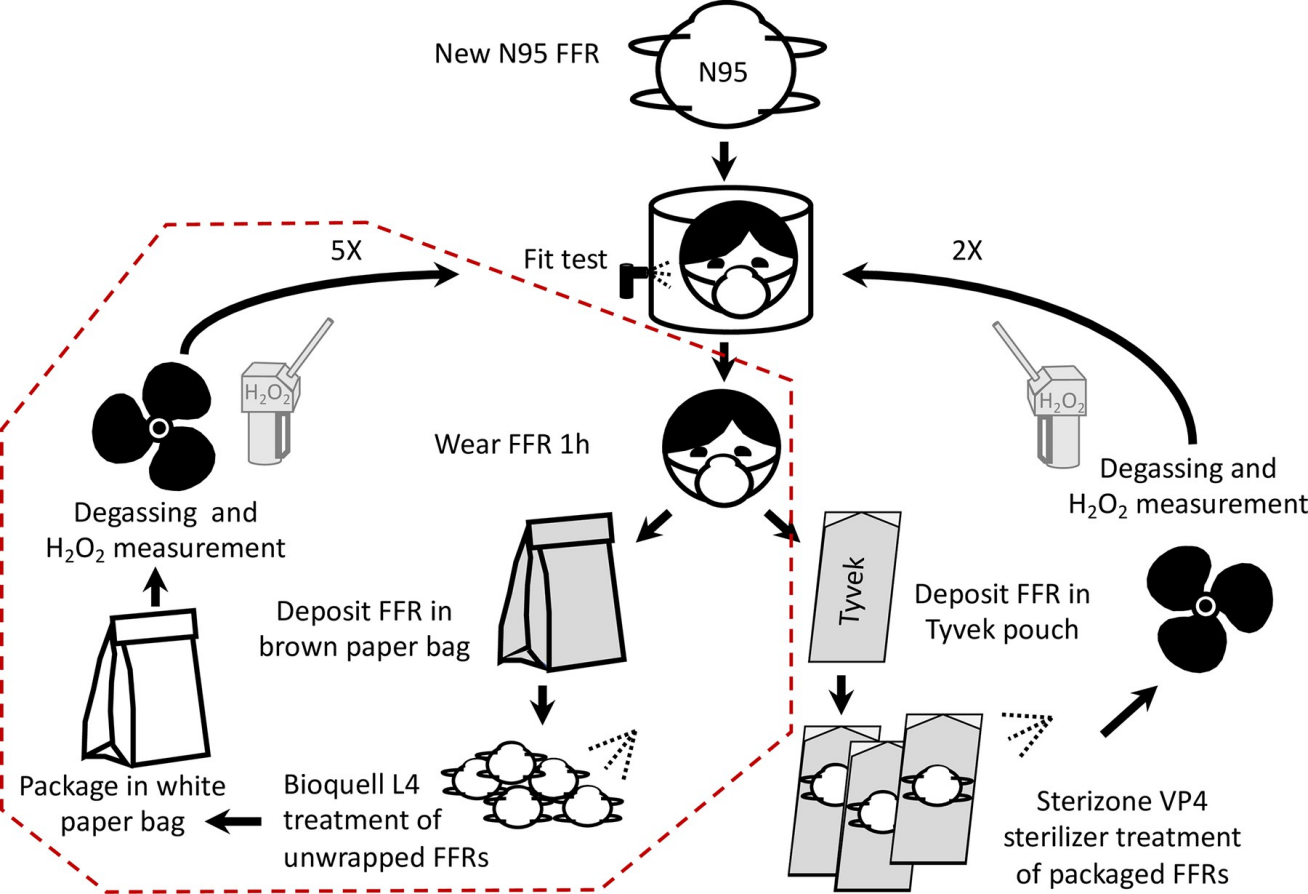
Schematic representation of the experimental design used with volunteers. The cycle used for large-scale implementation in the hospital is identified by the red dotted line.

The assay cycle started by conducting qualitative fit tests for new FFRs (Fig 2) according to Health Canada guidelines. Qualitative fit tests were conducted for each individual and every FFR was marked with a wearer ID. Volunteers wore the same mask throughout the experiment. Fit tests were conducted by evaluating taste sensitivity after vaporization using denatonium benzoate or saccharin solution [35]. Volunteers then wore their FFRs for 1 h, which was representative of the FFRs wearing time on the clinical floor in the beginning of the pandemic. Volunteers were invited to resume their normal activities when wearing FFR. Next, they placed the used FFRs in either brown paper bags (Bioquell L4) or Tyvek^®^ pouches (Sterizone VP4). The type of bag or pouch was dependent on the treatment as each has different compatibility restrictions, as indicated by the manufacturer protocols. FFRs were treated with Bioquell L-4 after being removed from their bags and with Sterizone VP4 in their Tyvek^®^ pouches. After treatment, N95 FFRs were packaged in white paper bags (Bioquell L-4) or left in their Tyvek^®^ pouches (Sterizone VP4) for aeration (24 h) before the next round of fit testing. Volunteers took part in another fit test followed by wearing the treated FFRs and filling out a questionnaire to describe any discomfort after a 1-hour wear (S1 and S2 Files). The questionnaire included a list of symptoms (eye/nose/throat/bronchi/skin irritation, face redness, fatigue, dyspnea, headache, nausea, intoxication) that could be selected and a section for volunteers to describe any additional symptoms. When necessary, symptoms were confirmed by a physician. This study looked at the comfort of volunteers wearing treated N95 FFRs and not to the comfort of N95 FFRs in general.

Upon the first round of treatment and wear (n = 13 FFRs), volunteers presented side-effects (irritation) from wearing the FFRs. Adjustments were made to the treatment protocol to ensure no hydrogen peroxide remained on the masks. The FFRs were left untouched for 24 h in their Tyvek^®^ pouches before subsequent Sterizone VP4 (N95 FFR decontamination cycle) treatment or in brown paper bags before Bioquell L-4 treatment. This allowed the FFRs to dry and reduced excessive hydrogen peroxide solubilization in the moist mask during treatment. The treated FFRs were also stored for an extended time after treatment. Hydrogen peroxide emanation for each FFR was measured using the method described below for up to 4 days until undetectable levels (LOD = 0.1ppm) were reached. The FFRs were then sent back to volunteers for another round of fit testing and wearing.

In total, the FFRs were treated up to 2 times with Sterizone VP4 sterilizer and up to 5 times with Bioquell L-4, following Canada Health guidelines for FFR retreatment and reuse. The number of cycles was set before analyzing the results. The proportion of adequate fit results was not a decisive factor in selecting the number of cycles conducted in our experiments.

### Hydrogen peroxide emission measurements

The hydrogen peroxide emitted from treated N95 FFRs was measured as described by Warburton and Hilliker [36] using a PortaSense III probe equipped with hydrogen peroxide sensor model 00–1042 (Analytical technology, US). Briefly, treated FFRs were allowed to rest (aeration) for 24 h before the first measurement. The FFRs were then placed in a sealed plastic bag (18 cm x 19 cm) for 30 min, after which the probe was inserted into the bag to measure hydrogen peroxide levels. To obtain an accurate reading, the probe was allowed a two-minute stabilization period in the bag before the measurement was read. Hydrogen peroxide emissions were measured daily until a level of 0 ppm was recorded (LOD = 0.1 ppm).

### Large-scale implementation at IUCPQ-UL

**Fig 3:**
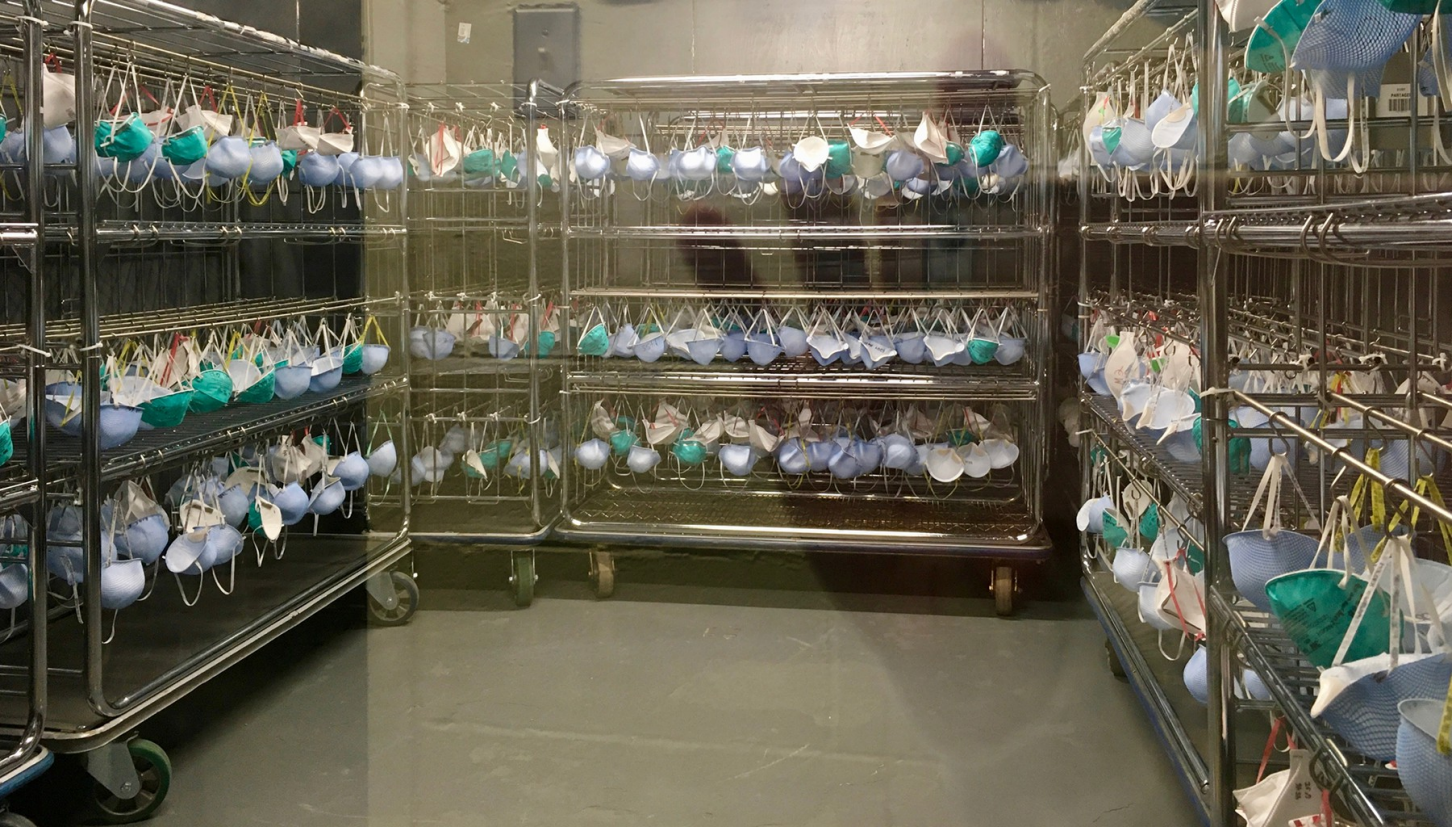
A 40-m^3^ decontamination room with 8 metal racks equipped with hooks, used for large-scale implementation of FFR treatment at the IUCPQ-UL.

The treatment selected for large-scale implementation at IUCPQ-UL is identified by the red dotted line in Fig 2. Dedicated collection bins were installed in COVID-19 units to collect used N95 FFRs. The FFRs were identified with the employee’s unit, last name, and employee number using permanent markers. N95 FFRs were individually packaged in brown paper bags that were also identified. They were then stored in cardboard boxes in a ventilated storage room until processing. After collection, FFRs were unwrapped and inspected. Masks with visual damage or deformation, improper identification, or visible soilage were discarded. The N95 FFRs were suspended from hooks by their elastic bands. Up to 960 N95 FFRs could be treated at the same time. Decontamination using Bioquell L-4 was conducted in a 40 m^3^ room that contained 8 metal racks (Fig 3).

During the study, the Bioquell L-4 was replaced by a Bioquell BQ-50, used in a 45 m^3^ room. Both devices use the same technology and only differ in their cycle customizability. The satisfactory results obtained using the Bioquell L-4 were therefore thought to also apply to the Bioquell BQ-50.

The Bioquell BQ-50 was programmed for a 90 m^3^ room cycle as recommended by the manufacturer for FFR treatment. Up to 960 FFRs were treated simultaneously. The decontamination cycle was validated using 20 biological indicators (HPV-Bi, Bioquell, PA, USA) containing 10^6^ spores of *Geobacillus stearothermophilus* (14 HPV-Bi were placed in the room, 6 of them between the FFRs). The indicators were processed according to the manufacturer’s instructions.

## Results

### Effect of treatment on new N95 FFRs

The filtration efficiencies presented in Table 1 are the minimum efficiencies measured on a filtration curve for NaCl particles that range from 20 nm to 600 nm, and with particle concentration measurements expressed in number of particles. When compared to untreated FFRs, treated FFRs do not have a reduced filtration efficiency or a notable variation in pressure drop.

**Table 1:** Minimum filtration performances measured at 85 L/min ^a^.

	Filtration efficiency (%) & pressure drop (mbar)		
**Treatments**	**No treatment (N = 1)**	**Sterizone VP4 (N = 3)**	**Bioquell L-4 (N = 8)**
**Moldex 1512**	96 %	94.3 ± 2.1 % 1.0 ± 0.1 mbar	-
	1.1 mbar		
**Moldex 1511**	97 %	-	97.0 ± 1.3 % 1.1 ± 0.1 mbar
	1.1 mbar		
**3M 1870+**	97 %	98.7 ± 0.6 % 0.7 ± 0.1 mbar	-
	0.8 mbar		

^a^ Raw data available in S1 Table

### Decontamination effectiveness

Bioquell biological indicators (10^6^ spores of *Geobacillus stearothermophilus*) were used to ensure proper decontamination with Sterizone VP4, Bioquell L-4 and Bioquell BQ-50. All the biological indicators that were placed inside the masks (folded into 3M 1870+ and held together between two Moldex FFRs) or in a separate pouch tested negative (absence of bacterial growth), indicating that a 6-log reduction of bacteria was achieved.

### Fit tests with volunteers

To ensure that the FFRs retain their integrity and remain airtight around their perimeter, fit tests were performed for the first use and between each decontamination cycle. All 23 new FFRs passed the first fit test (Table 2). At the end of the preselected number of cycles (twice with Sterizone VP4 or 5 times with Bioquell L-4), all FFRs and treatment combinations resulted in a noticeably altered fit. All models combined, 79% (11/14) of N95 FFRs passed the fit test after the second treatment (test 3) with Sterizone VP4. One hundred percent of N95 FFRs passed fit tests after 3 treatments with Bioquell L4, but only 38% (3/8) passed after 5 treatments. FFR deformation was the leading cause of fit test failures after treatment, and elastic band detachment was the second. There does not seem to be a notable difference between the types of FFR.

**Table 2:** Percentage of FFRs that passed fit tests after treatment.

Type of mask/treatment	Fit test 1 (new)^a^	Fit test 2	Fit test 3	Fit test 4	Fit test 5	Fit test 6
Moldex/Sterizone VP4	100% (8/8)	100% (8/8)	75% (6/8)	-	-	-
3M 1870+/Sterizone VP4	100% (6/6)	83% (5/6)	83% (5/6)	-	-	-
Moldex/Bioquell L-4	100% (5/5)	100% (5/5)	100% (5/5)	100% (4/4)^b^	100% (4/4)	50% (2/4)
3M 1870+/Bioquell L-4	100% (4/4)	100% (4/4)	100% (4/4)	100% (4/4)	50% (2/4)	25% (1/4)

^a^ Fit test performed on new N95 FFRs. The numbers in parentheses indicate the number of FFRs.

^b^ One FFR was discarded because the volunteer quit the study.

### Volunteer comfort

Volunteer discomfort and symptoms while wearing decontaminated FFRs were recorded via a self-assessment questionnaire. Following the first round of tests (n = 13 treated FFRs with Sterizone VP4) using both types of FFRs, 4 volunteers reported worrisome side-effects while wearing FFRs, including: skin irritation on the nose bridge, cheeks, and eyes (n = 2 wearing 3M masks), dry and itchy lips (n = 1 wearing 3M) and bronchial irritation (n = 1 wearing Moldex). This led us to reconsider the initial guidelines from Health Canada. We decided to add 24 h of drying in the bag or Tyvek^®^ pouch before treatment and extend the aeration period following treatment (in the bag or Tyvek^®^ pouch) until no hydrogen peroxide could be detected from each individual bagged FFR.

After this alteration to protocol, most volunteers did not experience the same negative effects. Only 1 volunteer (1/9) reported dry lips while wearing the 3M 1870+ treated with Sterizone VP4 and the Bioquell L-4, despite new aeration measures. However, volunteers reported different odors from FFRs that were treated using Sterizone VP4 compared to new N95 FFRs on the first (50%, 5/10) and second (78%, 7/9) cycles. Some volunteers liked the clean, chemical odor of decontaminated FFRs, while others found it unpleasant. No odors were reported for FFRs treated with Bioquell L-4. We observed no link between discomfort and number of treatments.

### Hydrogen peroxide vapors emitted from decontaminated FFRs

When treated with the Bioquell L-4 and the Sterizone VP4 sterilizer, Moldex series 1500 masks continued to release hydrogen peroxide for 1 to 2 days after treatment, while vapors were released for 3 to 4 days for 3M 1870+ FFRs. When treated with Bioquell BQ-50 and Bioquell L-4 combined with R-30 aerator, no hydrogen peroxide was detected on the FFRs 24 h post-treatment (Table 3) (LOD = 0.1 ppm). The R-30 aerator and BQ-50 contain active charcoal filters that remove hydrogen peroxide.

**Table 3:** Aeration time required to achieve FFRs hydrogen peroxide emissions below 0.1 ppm ^a^^,^^b^.

PPE	Sterizone VP4^1^	Bioquell L-4	Bioquell L-4 + R-30 aerator	Bioquell BQ-50
3M 1870+	3–4 days	3–4 days	1 day	1 day
Moldex series 1500	1–2 days	1–2 days	1 day	1 day

^a^ Treatment with Sterizone VP4 sterilizer only

^b^ Maximum and minimum number of days at which no hydrogen peroxide emissions were detected

#### Large scale implementation

Five batches of N95 FFRs were processed for decontamination (approximately 6000 FFRs) using Bioquell BQ-50. Approximately 25% of FFRs were discarded due to soilage, deformation, or improper identification, resulting in the decontamination and storage of approximately 4500 N95 FFRs.

## Discussion

### Decontamination effectiveness

In the COVID-19 pandemic context, it was imperative to choose a FFRs decontamination method effective against SARS-CoV-2. In this study, we assumed that decontamination methods effective against 10^6^ *G*. *stearothermophilus* spores would also be effective against SARS-CoV-2. Indeed, *Bacillus* spores are used to validate sterilization cycles with autoclave, hydrogen peroxide, ethylene oxide, and ionizing radiation [37]. Even if we did not have direct evidence to validate this assumption when we started this study, other studies have confirmed the effectiveness of various disinfection methods against SARS-CoV-2, including hydrogen peroxide vapor [38].

### Impact of N95 FFR decontamination

New FFRs were treated and tested for filtration effectiveness. For the 2 types of N95 FFRs tested (Moldex series 1500 and 3M 1870+), treated FFR performance was not noticeably different from untreated reference FFRs for all methods. These results are consistent with previous reports of FFR treatment using hydrogen peroxide [13, 16–21, 23, 25, 26, 28].

In addition, biological indicators added to decontamination cycles using the hydrogen peroxide confirmed the inactivation of microorganisms of up to 10^6^ bacterial spores. Hydrogen peroxide is therefore a viable decontamination agent for FFRs since it does not alter the filtration properties of the masks and allows for biological inactivation.

### Fit tests with volunteers

To be effective, FFRs must fit around the contours of the face and be properly sealed. To assess the integrity of this seal post-treatment, FFRs were worn and treated multiple times, and were challenged each time using a vaporized denatonium benzoate or saccharin solution (fit test). A large majority of FFRs retained a proper seal after two treatments. The higher number of treatment cycles conducted with Bioquell L-4 and the higher relative fit retention may seem to suggest this technology causes less damage to N95 FFRs. However, due to the pre-established number of cycles and the low number of FFRs tested, we cannot make this assumption and instead, attribute this trend to coincidence. In fact, when upscaling the process using Bioquell BQ-50, at least 25% of the masks were discarded during inspection before or after treatment due to severely altered physical integrity or soilage, which is a clear indication of the lack of robustness of disposable FFRs. The one or two masks that did not fit after using Sterizone VP4 (1/6, 2/8) may have been due to indelicate handling while wearing and treating them. The Tyvek^®^ pouches used for Sterizone VP4 treatment had a small opening that may have contributed to the occasional mask deformation that was observed. Furthermore, the various face shapes of the volunteers may have distorted the FFRs and made them less resistant to the handling and treatment process. We believe the inappropriate fit to be mostly caused by handling and poor resiliency of the PPEs, which are intended for single use. Due to the variability of our results, we recommend reusing FFRs for the lowest number of treatment cycles that still ensure availability to workers, and only once if possible.

These results are not completely consistent with previous reports that found the fit is maintained for 3–10 cycles using ionized hydrogen peroxide technology [17, 18, 39]. However, these studies used different FFR models and fit tests were conducted only once after repeated treatments. Moreover, FFRs were not worn several times, which might have positively affected the fit retention observed in those studies.

Other studies have been conducted in hospitals with extended use of FFRs (no decontamination) [22, 40–44]. Similar to our study, these authors observed an increase in fit test failures with the number of times they were reused and/or hours of wear.

### Volunteer comfort

While wearing the first round of treated FFRs, 4 volunteers (40%) reported irritation. We attribute this type of discomfort to the residual hydrogen peroxide found in the FFRs. As we present in Table 2, even after the 24-h aeration period, hydrogen peroxide vapors may still be emitted and additional venting is required before all the hydrogen peroxide dissipates. Ventilation with active charcoal filters can reduce the aeration time required. There are no official guidelines available for hydrogen peroxide emissions from PPEs. However, an exposure limit for the time-weighted average concentration of up to a 10-hour workday during a 40-hour workweek (TWA) has been set at 1 ppm [45]. Previous authors have suggested that emissions from PPEs should be below 0.2 ppm [36].

The first FFRs were worn and treated according to the guidelines offered by Health Canada. These guidelines were developed based on new masks and not those that have been used, which may explain the discrepancy observed here in terms of hydrogen peroxide emissions and our need to increase venting times. Masks that have already been used may remain damp, which could increase hydrogen peroxide absorption into the FFR. Therefore, we increased the aeration period before treatment to allow the FFRs to properly dry. According to our results, the FFRs should also be vented for at least 4 days after treatment.

Following these modifications, only one volunteer reported dry lips following the 1-hour wear. This shows a drastic reduction in reported side effects. Considering the urgency of PPE shortages, this very low risk was considered acceptable. Additional work may be needed to unequivocally ensure chemical safety if the intent is to reuse FFRs regularly.

### Large-scale implementation at IUCPQ-UL

When considering the implementation of a large-scale FFR treatment regime, one must take into account the particularities of the institution: the configuration of the sterilization unit, the availability of equipment, the activities conducted at the institution, the available space and staff, and the time required for treatment and equipment management. Following the satisfactory results for the 2 hydrogen peroxide treatment methods discussed above and the particularities of the institution, both Strerizone VP4 sterilizer and Bioquell L-4/BQ-50 were considered for use at IUCPQ-UL. These two methods require the collection of used FFRs in hospital units, the inspection and discard of FFRs with visual damage or deformation, improper identification or visible soilage, and the packaging and identification of the FFRs after treatment. Although these steps take the same amount of time for both methods, the decontamination times differ.

Our experiment showed that using Bioquell L-4, 960 FFRs could be treated in a 20-h cycle. Since 20 FFRs can be treated per cycle in the Sterizone VP4 sterilizer, it would take 48 h to treat the same number of FFRs using that method. Using a Sterizone VP4 sterilizer would allow for the continuous treatment of FFRs. If FFR consumption does not exceed 480 per day, one dedicated Sterizone VP4 sterilizer that is run for 24 h/24 h could treat all the FFRs. However, using a Bioquell L-4/BQ-50 would require that the used FFRs are collected and stored until enough FFRs have been amassed for a cycle, meaning there would need to be a larger number of FFRs in circulation to avoid shortages.

After careful consideration, the IUCPQ-UL decided to use the Bioquell L-4/BQ-50 for the large-scale implementation of PPE treatment. This method was selected because only one Sterizone VP4 low-temperature sterilizer was available in the institute, which meant it could not be exclusively used for FFR treatment due to its required use for other hospital functions. Moreover, the Sterizone VP4 low-temperature hydrogen peroxide sterilizer was located in a 2-way clean area in the IUCPQ-UL sterilization unit. Introducing contaminated or used protective equipment into the clean area poses a risk to other hospital activities due to possible cross-contamination. On the other hand, the Bioquell L-4 was devoted to research center activities. Since most research activities were on pause because of the pandemic, the apparatus as well as research center staff and space, were available for FFRs decontamination.

### Study limitations

This study was intended as a proof of concept and to develop an effective protocol for FFR decontamination in case of emergency shortages. A few aspects of this study reduce its universality and may require additional investigation. First, only two mask models were tested here (Moldex series 1500 and 3M model 1870+). Other manufacturers or models may use different materials or designs that may influence the impact of the decontamination process on the FFRs. For instance, hydrogen peroxide treatment is incompatible with paper or other cellulose base material [1]. Other studies have concluded that hydrogen peroxide vapor provides efficient decontamination and does not alter the filtration capability of several FFRs models, namely: Maplewood, Delta Plus model PFF_2_ as well as 3M models 1860, 8211, and 9210 [7, 16, 18, 26–29]. Therefore one can reasonably assume that hydrogen peroxide vapor can be used with several FFRs models that do not contain cellulose or valves since the latter were not tested. Studies on extensive use and reuse of disposable FFRs reported various fit test failure rates and recommended great care in seal checks before use [22, 40]. Therefore, one can also reasonably assume that our conclusions about the lack of robustness of disposable Moldex series 1500 and 3M model 1870+ after repeated wearing and decontamination could apply to other FFRs models. In this study, we did not observe a significant difference in the fit of a molded model (5 sizes) with a nonadjustable nose bridge and a one-size model folded in 3 parts with an adjustable nose bridge after use and treatment.

This study was also conducted on a relatively small number of masks and wearers due to FFR shortages, and the FFRs were worn for only one hour at each cycle. However, the discomforts reported by our volunteers with standard post-treatment aeration time, as approved by FDA and Health Canada, are concerning. Institutions that want to decontaminate FFRs should consider extending the aeration time before and after treatment. A blind study could also be conducted in future research to address the subjectivity of self-reported discomforts. However, volunteers may be more likely to report discomforts when they are aware that they are wearing a treated FFR. The absence of adverse effects reported after the venting protocol was modified suggests that the lack of volunteer blinding in our study was not critical. The effectiveness of disinfection was also not directly assessed in the matrix of the FFRs, but rather through stand-alone biological indicators (BI) since the masks could not be damaged if they were to be reused. However, considering BI placement between 2 FFRs and the large concentration of inactivated bacterial spores, it is reasonable to assume that the FFRs were thoroughly permeated by hydrogen peroxide.

### Ethical considerations

The uncertainty of the pandemic, the rapid increase in hospital admissions, and PPE shortages cause stressful situations for healthcare personnel. The implementation of FFR treatment and reuse must take into account the wearers of these masks and their thoughts on this process. A study conducted during the pandemic revealed that only 47% of health care workers are comfortable with the reuse practices of disposable FFRs [46]. As for any clinical protocol, success relies on the trust, acceptability, and compliance of the subjects. Ensuring proper filtration characteristics, fit, and the absence of toxic residue is merely the first step of the process.

Several institutions have decided to use disposable FFR decontamination to allow multiple users for the same FFR [15, 29, 30]. This means that the FFRs can be worn, decontaminated, and worn again by a different user. However, Levine and collaborators have demonstrated that 3M 1870+ FFRs (and possibly other folded FFR models) have significantly reduced fit test success with a second user [19]. Since health care workers have reported poor face seal integrity after FFR reuse [22], the decontamination of FFRs for multiple users must be conducted with extreme caution, preferably in low-risk areas, and be accompanied by regular thorough fit testing [41, 43].

Since we did not use the treated FFRs in actual daily work settings, we cannot address the real-life implementation of this protocol. However, we have identified a few critical points that should be considered when contemplating FFR reuse:

- The workers need to be informed of every step in the treatment process to build trust. Training and clear communication are crucial.
- The difference in odor between new and treated FFRs needs to be discussed to avoid surprises and limit suspicion.
- Shortages can occur for specific FFR models and sizes, therefore not all workers will need retreated FFRs at the same time. A contingency plan must be in place to determine when retreated FFRs have to be distributed.
- The contingency plan must also consider that all workers might not have available FFRs. Indeed, we observed that 25% of the FFRs were rejected during inspection before and after retreatment (visible soilage, deformation, or improper identification). Moreover, new personnel coming in might not have enough retreated FFRs to use. An alternative must be in place for those workers.
- If an alternative mask is available, all the workers might prefer that to the retreated FFRs. The decision-making process for these situations should be determined.

## Conclusion

In this study, we demonstrated that Moldex series 1500 FFRs and 3M 1870+ FFRs are compatible for reuse after hydrogen peroxide treatment. Both technologies (Sterizone VP4 and Bioquell L-4/BQ-50) produced satisfactory results in this context. Based on previous studies on N95 FFR decontamination [23] and our results, we conclude that the limitations in N95 FFR treatment using hydrogen peroxide treatment lie primarily in the FFRs being damaged during wearing and handling, and less in the cumulative effects of the treatments themselves. This study has documented fit tests, filtration efficiency, and side effects for volunteers after wearing treated FFRs. We highlighted that 24-h aeration post-treatment is not sufficient to avoid sides effects for users. In addition, we have proposed a large-scale treatment protocol that allows for FFR tracing in hospitals during PPE shortages.

Our work also highlights the limitations of protocols developed outside of clinical settings and the importance of "real-word" testing in different settings. Health care personnel facing a pandemic are under pressure, making the reliability and safety of FFR disinfection and reuse a crucial factor in diminishing stress and ensuring compliance among personnel. This can be achieved, in part, by taking measures to reduce any discomfort that may arise when reusing FFRs, but also by ensuring proper communication and training.

## Supporting information

S1 File Self report form for volunteers wearing treated FFRs, French version approved by IUCPOQ-UL ethical committee. (DOCX)

S2 File Self report form for volunteers wearing treated FFRs, English translation. (DOCX)

S1 Table Raw data used to create Table 1. (XLSX)
