# Supplementary material for: Filtration performance, fit test and side effects of respiratory personal protective equipment following decontamination: Observations for user safety and comfort
Source: PLoS One. 2023 Jan 23;18(1):e0280426. doi: 10.1371/journal.pone.0280426 (PMC9870121; doi:10.1371/journal.pone.0280426)
Supplement: S1 File — (DOCX) [file pone.0280426.s001.docx]

**Titre du projet** «N95 : Test de décontamination et réutilisations répétées avec validation des taux de pénétration et test d’ajustement sur des volontaires dans un contexte de traçabilité des masques»

**Section A (À remplir avant le port du masque)**

Date :____________

No du masque :______________

Vous sentez-vous bien aujourd’hui? Oui / Non

Ressentez-vous des symptômes parmi les suivants?

Nez qui coule ou congestion nasale

Toux

Mal de gorge

Irritation bronchique

Fièvre

**Section B (À remplir après le port du masque)**

Remarquez-vous une odeur différente d’un masque neuf? Oui / Non

Si oui, SVP décrivez

________________________________________________________________________________________________

Avez-vous ressenti de l’inconfort lors du port de votre masque? Si oui, cochez toutes les cases applicables

Irritation des yeux

Irritation du nez

Irritation de la gorge

Irritation des bronches

Irritation de la peau

Rougeur au visage

Fatigue

Dyspnée

Mal de tête

Nausée

Intoxication

Autre, SVP précisez________________________________
