# Supplementary material for: Filtration performance, fit test and side effects of respiratory personal protective equipment following decontamination: Observations for user safety and comfort
Source: PLoS One. 2023 Jan 23;18(1):e0280426. doi: 10.1371/journal.pone.0280426 (PMC9870121; doi:10.1371/journal.pone.0280426)
Supplement: S2 File — (DOCX) [file pone.0280426.s002.docx]

**Project title**“N95: Decontamination and repeated used, with validation of filtration efficiency and fitting with volunteers in a trackability context. “

**Section A (To complete before wearing the N95)**

Date:____________

FFR number:______________

Do you feel good today? Yes / No

Do you have symptoms among the following?

Runny nose or nasal congestion

Chough

Sore throat

Bronchial irritation

Fever

**Section B (to fil after wearing FFR)**

Did you notice a smell different from new FFR? Yes / No

If yes, please describe

________________________________________________________________________________________________

Did you fell discomfort when wearing your FFR? If yes, please check all those that apply

Yes irritation

Nose irritation

Sore throat

Bronchial irritation

Skin irritation

Redness in the face

Fatigue

Dyspnea

Headache

Nausea

Intoxication

Other, please precise________________________________
